# Cardiovascular events in delayed presentation of HIV: the prospective PISCIS cohort study

**DOI:** 10.3389/fmed.2023.1182359

**Published:** 2023-06-21

**Authors:** Raquel Martín-Iguacel, Mari Carmen Vazquez-Friol, Joaquin Burgos, Andreu Bruguera, Juliana Reyes-Urueña, Sergio Moreno-Fornés, Jordi Aceitón, Yesika Díaz, Pere Domingo, Maria Saumoy, Hernando Knobel, David Dalmau, Beatriz Borjabad, Isik Somuncu Johansen, Jose M. Miro, Jordi Casabona, Josep M. Llibre

**Affiliations:** ^1^Centre of Epidemiological Studies of HIV/AIDS and STI of Catalonia (CEEISCAT), Health Department, Generalitat de Catalunya, Badalona, Spain; ^2^Department of Infectious Diseases, Odense University Hospital, Odense, Denmark; ^3^Infectious Diseases Unit, University Hospital of Ferrol, A Coruña, Spain; ^4^Department of Infectious Diseases, Hospital Universitari de la Vall d'Hebron, Barcelona, Spain; ^5^CIBER Epidemiologia y Salud Pública (CIBERESP), Madrid, Spain; ^6^Department of Paediatrics, Obstetrics and Gynecology and Preventive Medicine, Universitat Autònoma de Barcelona, Badalona, Spain; ^7^Infectious Diseases Unit, Hospital Universitari de la Santa Creu i Sant Pau, Barcelona, Spain; ^8^Department of Internal Medicine and Infectious Diseases, Hospital Universitari de Bellvitge, Hospitalet de Llobregat, Barcelona, Spain; ^9^Department of Infectious Diseases, Hospital del Mar- Parc de Salut MAR, Barcelona, Spain; ^10^Department of Internal Medicine, Hospital Universitari Mútua Terrassa, Barcelona, Spain; ^11^Department of Internal Medicine, Consorci Sanitari Integral, Hospitalet del Llobregat, Barcelona, Spain; ^12^Hospital Clínic-Institut d'Investigacions Biomèdiques August Pi i Sunyer, University of Barcelona, Barcelona, Spain; ^13^Centro de Investigación Biomédica en Red Enfermedades Infecciosas (CIBERINFEC), Instituto de Salud Carlos III, Madrid, Spain; ^14^Fundació Institut D'investigació en Ciències de la Salut Germans Trias I Pujol (IGTP), Badalona, Spain; ^15^Infectious Diseases Department, University Hospital Germans Trias i Pujol, Barcelona, Spain; ^16^Fight Infections Foundation, Barcelona, Spain

**Keywords:** late HIV presentation, cardiovascular disease, myocardial infarction, cerebrovascular disease, HIV

## Abstract

**Objectives:**

People with HIV (PWH) have a higher cardiovascular risk than the general population. It remains unclear, however, whether the risk of cardiovascular disease (CVD) is higher in late HIV presenters (LP; CD4 ≤ 350 cells/μL at HIV diagnosis) compared to PWH diagnosed early. We aimed to assess the rates of incident cardiovascular events (CVEs) following ART initiation among LP compared to non-LP.

**Methods:**

From the prospective, multicentre PISCIS cohort, we included all adult people with HIV (PWH) initiating antiretroviral therapy (ART) between 2005 and 2019 without prior CVE. Additional data were extracted from public health registries. The primary outcome was the incidence of first CVE (ischemic heart disease, congestive heart failure, cerebrovascular, or peripheral vascular disease). The secondary outcome was all-cause mortality after the first CVE. We used Poisson regression.

**Results:**

We included 3,317 PWH [26 589.1 person/years (PY)]: 1761 LP and 1556 non-LP. Overall, 163 (4.9%) experienced a CVE [IR 6.1/1000PY (95%CI: 5.3–7.1)]: 105 (6.0%) LP vs. 58 (3.7%) non-LP. No differences were observed in the multivariate analysis adjusting for age, transmission mode, comorbidities, and calendar time, regardless of CD4 at ART initiation [aIRR 0.92 (0.62–1.36) and 0.84 (0.56–1.26) in LP with CD4 count <200 and 200– ≤ 350 cells/μL, respectively, compared to non-LP]. Overall mortality was 8.5% in LP *versus* 2.3% in non-LP (*p* < 0.001). Mortality after the CVE was 31/163 (19.0%), with no differences between groups [aMRR 1.24 (0.45–3.44)]. Women *vs*. MSM and individuals with chronic lung and liver disease experienced particularly high mortality after the CVE [aMRR 5.89 (1.35–25.60), 5.06 (1.61–15.91), and 3.49 (1.08–11.26), respectively]. Sensitivity analyses including only PWH surviving the first 2 years yielded similar results.

**Conclusion:**

CVD remains a common cause of morbidity and mortality among PWH. LP without prior CVD did not exhibit an increased long-term risk of CVE compared with non-LP. Identifying traditional cardiovascular risk factors is essential for CVD risk reduction in this population.

## Introduction

Despite absolute rates of cardiovascular disease (CVD) declining significantly over time, people with HIV (PWH) continue to have an ~50% higher relative risk compared with HIV-negative individuals (1). The American Heart Association recommends adjusting the calculated risk estimate upward by 1.5–2 times for PWH on the basis that most risk calculators tend to underestimate their cardiovascular risk (2).

Late HIV diagnosis, defined as CD4 count < 350 cells/μL or AIDS at diagnosis regardless of CD4 count, still represents an unmet need, with half of PWH being diagnosed late. Individuals not belonging to the traditional HIV risk groups, such as heterosexual men, females, older individuals, and migrants, are at a higher risk of late HIV diagnosis (3). Older people are at increased risk of both late HIV diagnosis and CVD, and their proportional rates among PWH are increasing (4).

Late HIV diagnosis has been associated with increased risks of death, AIDS, and non-AIDS comorbidities (5, 6). The risk for AIDS and death is highest in the first year, mainly due to AIDS-related conditions, often present at baseline (3). Factors such as unremitting inflammation potentially related to higher HIV reservoirs and persistent immune activation due to late ART initiation (7), have been associated with premature aging and may predispose to non-AIDS comorbidities (8). However, their link with CVD in this scenario has not been proven, and the understanding of the subsequent CV risk in late presenters (LPs) is incomplete. Furthermore, additional factors other than low CD4 cell count and chronic inflammation may also contribute to the disparity in long-term morbidity and mortality in LP such as differences in access and adherence to care (9, 10), behavioral factors such as smoking (11), the burden of comorbidities, and socio-economic factors. In addition, lower CD4 count led to more conservative surgical approaches to CV complications (12).

While some studies have not found a greater risk of CVD in LP (13–15), others have reported that CD4 count depletion along the course of the HIV disease is associated with a higher risk of CVD. The latter, however, has been mainly described in immunological non-responders after the initiation of ART or in individuals with unsuppressed viremia (16–20).

It is thus uncertain whether the long-term cardiovascular risk in LP who initiate effective ART is higher than in those individuals who were diagnosed early. We aimed to assess the rates of incident CV events following ART initiation among LP in a prospective cohort.

## Methods

### Study design and data sources

This is a cohort study conducted in Catalonia, Spain. On January 2021, Catalonia had a population of 7.7 million citizens and an estimated adult prevalence of HIV infection of 0.4%. The Catalan healthcare system provides universal, tax-funded healthcare, and ART to all citizens.

Data were retrieved from the PISCIS cohort (Catalonian and Balearic Islands HIV cohort) and PADRIS (Public Data Analysis for Health Research and Innovation Program). Briefly, PISCIS is an ongoing, prospective, multicentre, population-based cohort that includes all PWH aged ≥16 years followed in one of the 16 collaborating hospitals, representing 84% of all PWH in Catalonia, from 1998 to 2022 (21). Data are updated yearly and include demographics, date of HIV diagnosis, AIDS-defining events, ART, and measurement of CD4 count and plasma HIV-RNA over time. PADRIS is a central research-oriented database that gathers and cross-matches real-world health data generated by the different public health systems (SISCAT), provided by the Catalan Agency for Health Quality and Evaluation (AQuAS). The database includes comorbidity data from hospital discharge diagnoses and primary healthcare from 2005 according to the International Classification of Disease 10th revision (ICD-10) (22). We used ICD-10 codes to define comorbidities according to the Swedish National Study of Aging and Care in Kungsholmen (SNAC-K) cohort (www.snac-k.se). Mortality data were obtained by cross-matching the information from PISCIS, PADRIS, and the national mortality registry.

The study complies with the STROBE reporting guidelines.

### Ethics approval

The PISCIS cohort has received ethical approval from Germans Trias i Pujol University Hospital's Clinical Research Ethics Committee, reference number EO-11-108, and patient data extraction is allowed by the 203/2015 Decree from the Catalan Health Department. All data are pseudo-anonymized in accordance with Regulation 2016/679 of the European Parliament.

### Study population

We included all treatment naïve PWH aged ≥18 years, who initiated ART between 1 January 2005 and 30 June 2019. PWH with prior CV events were excluded from the analysis.

Individuals were classified according to their CD4 count at ART initiation into LP (i.e., CD4 count < 200 and 200–350 cells/μ) and non-LP (i.e., CD4 count >350 cells/μL). Non-LP contributed as the reference population.

### Outcomes

The primary outcome was the first CV event after ART initiation, defined as the first event of ischemic heart disease, congestive heart failure, cerebrovascular disease, or peripheral vascular disease during the study period.

The secondary outcome was all-cause mortality among PWH experiencing a CV event.

### Statistical analysis

Continuous variables were described as the median and interquartile range (IQR), whereas categorical variables were presented as the frequency and percentage over available data. We used Pearson's chi-squared test, *t*-test, and Mann–Whitney U-test to compare these variables between LP and non-LP. For polychotomous categorical variables with a statistically significant chi-square test of homogeneity, we further provided a significance test of the difference between proportions across categories of the variable between LP and non-LP.

Individuals were followed from ART initiation to the first CV event, loss-to-follow-up, death, or end of the observation period (30 June 2021), whichever occurred first. The cumulative incidence of the first CV event in LP *vs*. non-LP was compared using the log-rank test. We used Poisson regression to assess the association between CD4 count at ART initiation (< 200, 200–350, and >350 cells/μL) and the risk of a subsequent CV event. We adjusted for the following potential confounders at baseline: age (time-updated), gender, mode of HIV transmission, region of birth, time-updated calendar period [2005–2009, 2010–2014, 2015–2019, according to pre-integrase strand transfer inhibitor (INSTI)-available period, INSTI-available period, and universal ART implementation, respectively] (23), HIV-1 viral load, AIDS-defining disease, comorbidities (diabetes mellitus, arterial hypertension, dyslipidemia, chronic kidney, lung, or liver disease, malignancy, or depression), and educational level. We provide incidence rates (IRs) and IR ratios (IRRs) and their corresponding 95% confidence interval (CI).

For mortality after the first CV event, individuals were followed from the CV event to death, loss-to-follow-up, or 30 June 2021, whichever came first. The cumulative mortality was compared using the log-rank test. We used a Poisson regression analysis to assess the risk of late HIV presentation and mortality adjusting for the above-mentioned potential confounders. We provide mortality rates (MRs) and MR ratios (MRRs) and a 95%CI.

We conducted a sensitivity analysis including only PWH who initiated ART and survived 2 years, in order to remove the bias of the increased risk of severe AIDS-related comorbidities and complications in the initial period and to assess the effect of early immune recovery on CVD (3, 24). PWH were stratified according to their 2-year CD4 count (< 200, 200–500, and >500 cells/μL in LP and ≤ 500 cells/μL and >500 cells/μL in non-LP).

All analyses were conducted with STATA software (v.16; Stata Corp, College Station, TX, USA).

## Results

### Study participants

**Figure 1:**
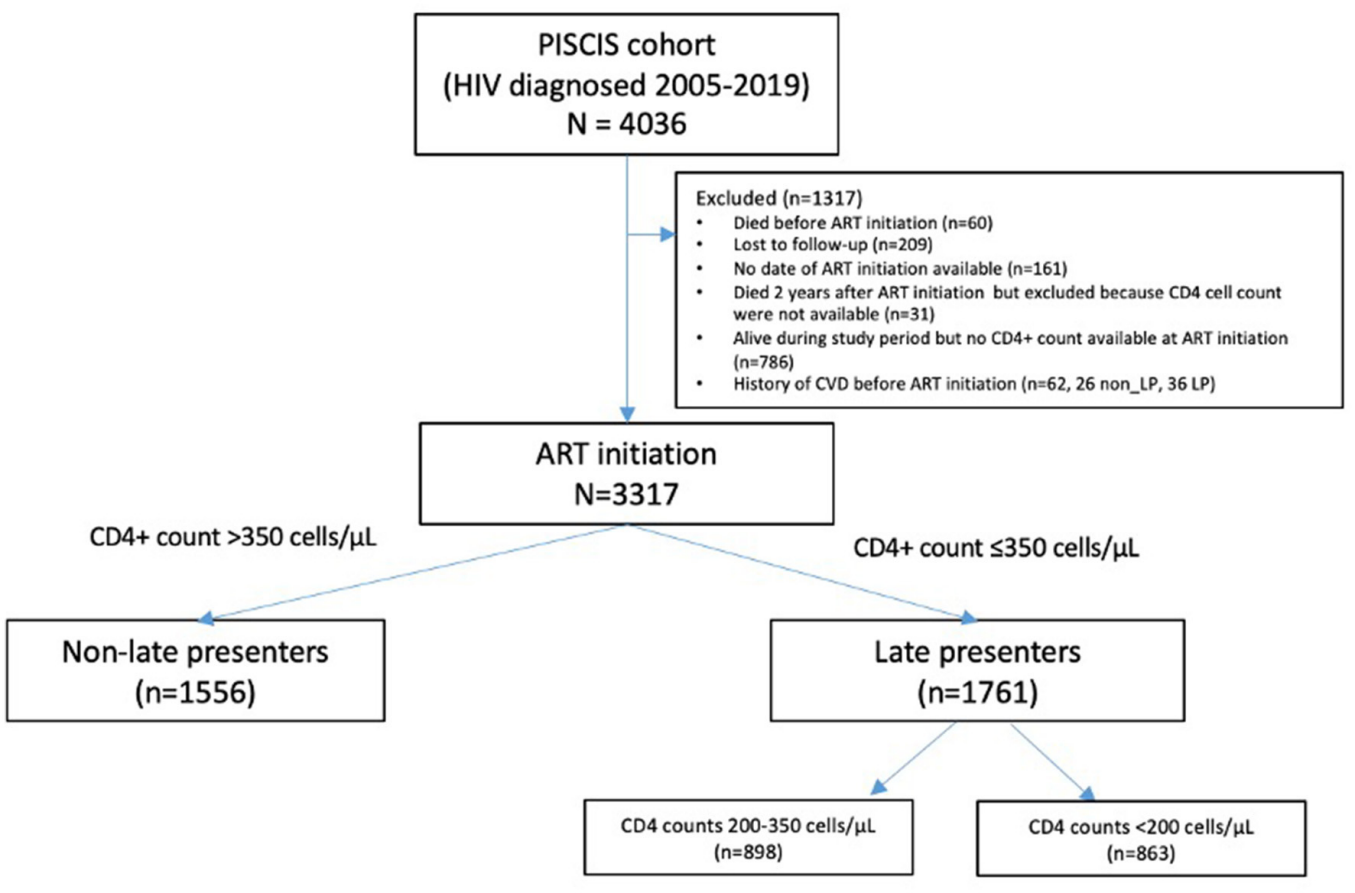
Flowchart of cohort construction.

We identified 4036 adult PWH who initiated ART between 2005 and 2019. Of them, 3317 met the inclusion criteria, 1556 (46.9%) non-LP, and 1,761 (53.1%) LP (Figure 1). The median follow-up was 8.0 years (IQR: 5.0–11.2), giving rise to 26 589.1 persons/years (PY) at risk.

Table 1 summarizes baseline characteristics. LP were more likely heterosexual men, women, IDU, older, treated with protease inhibitors or non-nucleoside reverse transcriptase inhibitors, having higher plasma HIV-RNA, and having lower educational levels. LP had higher rates of AIDS-defining events, liver disease, dementia, and less arterial hypertension.

**Table 1:** Baseline characteristics of the study participants.

**Study population according to initial CD4 cell count at ART**				**Study population according to immune recovery 2 years after ART initiation**				
	**Non-LP** ***n*** = **1,556 (46.9%)**	**LP** ***n*** = **1,761 (53.1%)**	***P*** **-value**	**LP** ***n*** = **1,386**				**Non-LP CD4** >**500 cells/**μ**L** ***n*** = **1,114 (89.9%)**
				**CD4 cell count (cells/**μ**)**				
				>**500** ***n*** = **627 (45.2%)**	>**350-500** ***n*** = **361 (26.0%)**	≥**200-350** ***n*** = **286 (20.6%)**	<**200** ***n*** = **112 (8.1%)**	
Male, *n* (%)	1,360 (87.4)	1,468 (83.4)	0.003	539 (86.0)	305 (84.5)	226 (79.0)	94 (83.9)	995 (89.3)
Age (years), median (IQR)	34 (29–41)	38 (31–45)	< 0.001	36 (30–42)	38 (32–45)	40 (34–47)	39 (34–47)	35 (29–41)
Birth area, *n* (%)			0.010					
Spain	702 (57.1)	867 (59.3)	0.018	326 (60.5)	174 (57.1)	149 (63.7)	62 (67.4)	514 (57.9)
Europe	157 (12.8)	130 (8.9)	0.006	47 (8.7)	30 (9.8)	15 (6.4)	2 (2.2)	109 (12.3)
Africa	54 (4.4)	86 (5.9)	0.043	17 (3.2)	19 (6.2)	22 (9.4)	10 (10.9)	36 (4.1)
America	297 (24.2)	360 (24.6)	0.33	139 (25.8)	79 (25.9)	48 (20.5)	16 (17.4)	215 (24.2)
Asia	19 (1.6)	19 (1.3)	0.70	10 (1.9)	3 (1.0)	0	2 (2.2)	14 (1.6)
Route of HIV transmission, *n* (%)			< 0.001					
MSM	1,088 (69.9)	890 (50.5)	< 0.001	410 (65.4)	194 (53.7)	93 (32.5)	34 (30.4)	823 (73.9)
Heterosexual men	150 (9.6)	322 (18.3)	< 0.001	82 (13.1)	61 (16.9)	78 (27.3)	28 (25.0)	103 (9.3)
Women	157 (10.1)	233 (13.2)	0.005	74 (11.8)	48 (13.3)	51 (17.8)	12 (10.7)	99 (8.9)
IDU	78 (5.0)	180 (10.2)	< 0.001	26 (4.2)	37 (10.3)	32 (11.2)	28 (25.0)	43 (3.9)
Unknown	83 (5.3)	136 (7.7)	0.006	35 (5.6)	21 (5.8)	32 (11.2)	10 (8.9)	46 (4.1)
**First ART- regimen in the first 2 years**, ***n*** **(%)**								
NNRTI-based regimen	597 (38.4)	786 (44.6)	< 0.001	329 (52.5)	181 (50.1)	127 (44.4)	45 (40.2)	475 (42.6)
PI-based regimen	370 (23.8)	698 (39.6)	< 0.001	226 (36.0)	149 (41.3)	130 (45.5)	66 (58.9)	273 (24.5)
INSTI-based regimen	680 (43.7)	562 (31.9)	< 0.001	193 (30.8)	102 (28.3)	76 (26.6)	36 (32.1)	492 (44.2)
**CD4 count at ART initiation (cells/**μ**L)**, ***n*** **(%)**								
< 100 cells/μL	-	472 (26.8)		65 (18.2)	89 (24.9)	125 (34.9)	79 (22.1)	-
100–199 cells/μL	-	391 (22.2)		106 (35.3)	94 (31.3)	82 (27.3)	18 (6.0)	-
200–350 cells/μL	-	898 (51.0)		456 (62.6)	178 (24.5)	79 (10.9)	15 (2.1)	-
>350–500 cells/μL	725 (46.6)	-		-	-	-	-	497 (44.6)
>500 cells/μL	831 (53.4)	-			-	-	-	617 (55.4)
Viral load at ART initiation (log^10^ copies/mL), median (IQR)	4.4 (3.6–4.9)	4.9 (4.0–5.5)	< 0.001	4.9 (4.3–5.3)	4.8 (3.9–5.5)	4.9 (4.0–5.7)	4.9 (4.0–5.7)	4.4 (3.7–4.9)
CD4 cell count 2 years after ART initiation (cells/μL), median (IQR)	804 (633–999)	477 (329–630)	< 0.001	-	-	-	-	-
Detectable viral load 2 years after ART initiation (>200 copies/mL), *n* (%)	59 (5.2)	89 (6.8)	0.11	25 (4.2)	14 (4.1)	23 (8.6)	27 (25.0)	35 (3.5)
History of AIDS-defining events at ART initiation, n (%)	25 (1.6)	227 (12.9)	< 0.001	52 (8.3)	44 (12.2)	54 (18.9)	37 (33.0)	14 (1.3)
**Comorbidity at ART initiation**, ***n*** **(%)**								
Chronic kidney disease	9 (0.6)	8 (0.5)	0.62	3 (0.5)	5 (1.4)	2 (0.7)	0	11 (1.0)
Chronic liver disease	64 (4.1)	114 (6.5)	0.003	42 (6.7)	36 (1.0)	36 (12.6)	24 (21.4)	63 (5.7)
Chronic lung disease^a^	14 (0.9)	23 (1.3)	0.26	10 (1.6)	4 (1.1)	9 (3.2)	1 (0.9)	17 (1.5)
Diabetes	13 (0.8)	15 (0.9)	0.96	9 (1.4)	7 (1.9)	6 (2.1)	3 (2.7)	14 (1.3)
Arterial hypertension	123 (9.9)	102 (7.4)	0.019	44 (7.0)	30 (8.3)	24 (8.4)	4 (3.6)	115 (10.3)
Dyslipidaemia	63 (4.1)	56 (3.2)	0.18	26 (4.1)	9 (2.5)	11 (3.8)	4 (3.3)	58 (4.1)
Hematological neoplasm	5 (0.3)	11 (0.6)	0.21	4 (0.6)	6 (1.7)	4 (1.4)	5 (4.5)	8 (0.7)
Solid neoplasm	24 (1.5)	56 (3.2)	0.002	25 (4.0)	19 (5.3)	28 (9.8)	16 (14.3)	39 (3.5)
Dementia	1 (0.08)	5 (0.3)	0.14	2 (0.3)	1 (0.3)	3 (1.1)	3 (2.7)	1 (0.09)
Depression	102 (6.6)	94 (5.3)	0.14	53 (8.5)	25 (6.9)	33 (11.5)	11 (9.8)	115 (10.3)
Modified Charlson Comorbidity Score			0.004					
at ART initiation^b^, *n* (%)								
0	1,442 (92.7)	1,569 (89.1)	< 0.001	583 (92.3)	329 (89.7)	256 (88.0)	95 (78.5)	1,325 (92.9)
1	76 (4.9)	118 (6.7)	0.026	28 (4.4)	21 (5.7)	24 (8.3)	17 (14.1)	68 (4.8)
2–3	32 (2.1)	62 (3.5)	0.011	19 (3.0)	14 (3.8)	10 (3.4)	8 (6.6)	28 (2.0)
≥4	6 (0.4)	12 (0.7)	0.25	2 (0.3)	3 (0.8)	1 (0.3)	1 (0.8)	5 (0.4)
Calendar time of ART initiation, *n* (%)			< 0.001					
2005–2009	216 (24.1)	680 (75.9)	< 0.001	229 (36.6)	147 (41.0)	144 (51.1)	58 (51.8)	138 (12.5)
2010–2014	691 (53.4)	517 (46.6)	0.001	261 (41.8)	138 (38.4)	83 (29.4)	35 (31.3)	562(52.1)
2015–2019	649 (57.5)	479 (42.5)	< 0.001	135 (21.6)	74 (20.6)	55 (19.5)	19 (17.0)	404 (36.6)
Educational level, *n* (%)			< 0.001					
None or primary education only	274 (23.6)	434 (35.7)	< 0.001	132 (28.2)	86 (33.0)	84 (43.8)	29 (44.6)	179 (21.0)
Secondary education	467 (40.2)	444 (36.5)	0.063	185 (39.5)	104 (39.9)	60 (31.3)	20 (30.8)	353 (41.3)
University	420 (36.2)	338 (27.8)	< 0.001	151 (32.3)	66 (25.3)	42 (21.9)	15 (23.1)	316 (37.0)

ART, antiretroviral therapy; CVD, cardiovascular disease (includes ischemic heart disease, congestive heart failure, cerebrovascular disease, peripheral vascular disease); IDU, Injection drug use; INSTI, integrase strand transfer inhibitor; IQR, interquartile range (percentiles 25th and 75th); LP, late presenters; non-LP, non-late presenters; MSM, men who have sex with men; NNRTI, non-nucleoside reverse transcriptase inhibitors; PI, protease inhibitor.

^a^Chronic lung disease included chronic obstructive pulmonary disease, lung emphysema, unspecified chronic bronchitis, and bronchiectasis.

^b^Described in Supplementary Table 9.

At 2 years, 112 (8.1%) LP remained with a CD4 count of < 200 cells/μL, 286 (20.6%) had 200–350 cells/μL, 661 (26.0%) had 351–500 cells/μL, and 627 (45.2%) had >500 cells/μL. LP with a 2-year CD4 count of < 200 cells/μL after ART initiation had higher rates of detectable HIV-RNA (*p* < 0.0001) (Table 1).

### CVD outcomes

Overall, 163 (4.9%) PWH experienced a first CV event during the study period [IR 6.1/1000 PY (95%CI: 5.3–7.1)]: 105 (6.0%) LP *vs*. 58 (3.7%) non-LP (p = 0.003). Forty-eight (29.4%) experienced a heart failure event, 42 (25.8%) experienced an ischemic heart event, 53 (32.5%) experienced a cerebrovascular event, and 20 (12.3%) experienced a peripheral artery event (Supplementary Table 3). Most PWH experienced 1 CV event (*n* = 136, 83.4%), 24 (14.7%) experienced 2 CV events, and 3 (1.8%) experienced 3 different events. The rates of CV events did not change along the calendar time analyzed (Table 2).

**Table 2:** Incidence rate of a first CV event in the overall cohort, including all LP and non-LP.

	**CV Events (*n*)**	**IR per 1,000 PY**	**IRR (95% CI)**	**aIRR (95%CI)^a^**
Total	163	6.1 (5.3–7.1)		
**Gender**				
Female	26	6.3 (4.3–9.3)	Ref (1)	
Male	137	6.1 (5.2–7.2)	0.97 (0.64–1.47)	
**Age (time-updated) (years)**				
< 40	29	2.4 (1.7–3.4)	Ref (1)	Ref (1)
40–49	55	6.1 (4.7–8.0)	2.58 (1.64–4.04)	2.11 (1.33–3.33)
50–59	54	13.1 (1.0–17.1)	5.49 (3.50–8.62)	3.87 (2.42–6.20)
≥60	25	19.1 (12.9–28.3)	8.04 (4.71–13.72)	4.56 (2.53–8.20)
**Country of birth**				
Spain	108	8.3 (6.8–1.0)	Ref (1)	
Europe	14	6.6 (3.9–11.2)	0.80 (0.46–1.40)	
Africa	8	6.6 (3.3–13.3)	0.80 (0.39–1.65)	
America	16	2.8 (1.7–4.6)	0.34 (0.20–0.58)	
Asia	1	3.6 (0.5–25.5)	0.43 (0.06–3.12)	
**Transmission mode**				
MSM	57	3.6 (2.8–4.7)	Ref (1)	Ref (1)
Heterosexual men	47	12.5 (9.4–16.6)	3.48 (2.37–5.12)	2.43 (1.62–3.67)
Women	16	4.7 (2.9–7.7)	1.32 (0.76–2.30)	1.14 (0.65–1.99)
IDU	28	13.7 (9.5–19.9)	3.82 (2.43–6.01)	2.23 (1.35–3.71)
Unknown/other	15	9.9 (5.9–16.4)	2.75 (1.56–4.85)	1.98 (1.11–3.55)
**CD4 cell count at ART initiation**				
Late presenters, CD4 ≤ 350 cells/μL	105	6.9 (5.7–8.3)	1.35 (0.98–1.86)	
Non-late presenters, Cd4 >350 cells/μL	58	5.1 (3.9–6.6)	Ref (1)	
**CD4 cell count at ART initiation**				
Late presenters, CD4 < 200 cells/μL	63	8.7 (6.8–11.2)	1.71 (1.19–2.44)	0.92 (0.62–1.36)
Late presenters, CD4 200– ≤ 350 cells/μL	42	5.3 (3.9–7.1)	1.03 (0.69–1.53)	0.84 (0.56–1.26)
Non-late presenters, Cd4 >350 cells/μL	58	5.1 (3.9–6.6)	Ref (1)	Ref (1)
**HIV viral load at ART initiation**				
>100,000 copies/ml	43	5.5 (4.1–7.4)	0.87 (0.61–1.23)	
≤ 100,000 copies/ml	120	6.4 (5.3–7.6)	Ref (1)	
**Calendar time (time-updated)**				
2005–2009	8	4.5 (2.2–9.0)	0.72 (0.35–1.48)	0.81 (0.39–1.69)
2010–2014	47	6.3 (4.8–8.4)	1.02 (0.72–1.44)	1.16 (0.82–1.64)
2015–2021	108	6.2 (5.1–7.5)	Ref (1)	Ref (1)
**AIDS-defining event at ART initiation**				
Yes	29	13.8 (9.6–19.9)	2.52 (1.69–3.77)	1.49 (0.95–2.34)
No	134	5.5 (4.6–6.5)	Ref (1)	Ref (1)
**Educational level** ^b^				
None or primary education only	55	9.5 (7.3–12.3)	Ref (1)	
Secondary education	33	4.5 (3.2–6.3)	0.47 (0.31–0.73)	
University	19	3.3 (2.1–5.1)	0.35 (0.21–0.58)	
**Comorbidities at ART initiation**				
**Diabetes**				
Yes	6	32.0 (14.4–71.3)	5.38 (2.38–12.17)	1.32 (0.51–3.39)
No	157	5.9 (5.1–7.0)	Ref (1)	Ref (1)
**Arterial hypertension**				
Yes	16	5.8 (4.9–6.8)	2.48 (1.48–4.15)	1.83 (1.07–3.11)
No	147	14.3 (8.8–23.3)	Ref (1)	Ref (1)
**Dyslipidaemia**				
Yes	13	16.5 (9.6–28.5)	2.84 (1.61–5.01)	1.34 (0.71–2.55)
No	150	5.8 (5.0–6.8)	Ref (1)	Ref (1)
**Chronic kidney disease**				
Yes	3	31.0 (1.0–96.1)	5.13 (1.64–16.08)	1.98 (0.58–6.76)
No	160	6.0 (5.2–7.1)	Ref (1)	Ref (1)
**Chronic lung disease**				
Yes	6	26.5 (11.9–59.0)	4.45 (1.97–1.06)	1.67 (0.71–3.90)
No	157	6.0 (5.1–7.0)	Ref (1)	Ref (1)
**Chronic liver disease**				
Yes	25	20.3 (13.7–30.1)	3.74 (2.44–5.72)	2.38 (1.48–3.82)
No	138	5.4 (4.6–6.2)	Ref (1)	Ref (1)
**Malignancy** ^c^				
Yes	11	22.4 (12.4–40.5)	3.85 (2.09–7.10)	2.27 (1.20–4.30)
No	152	5.8 (5.0–6.8)	Ref (1)	Ref (1)
**Depression**				
Yes	14	9.9 (5.9–16.8)	1.68 (0.97–2.90)	1.08 (0.61–1.91)
No	149	5.9 (5.0–6.9)	Ref (1)	

aIRR, adjusted incidence rate ratio; CVD, cardiovascular disease (includes ischemic heart disease, congestive heart failure, cerebrovascular disease, peripheral vascular disease); IR, incidence rate; IRR, incidence rate ratio; IDU, Injection drug use; MSM, men who have sex with men; PY, person-years.

^a^The aIRR was adjusted for age, transmission mode, CD4 cell count, calendar time and AIDS-defining event, and comorbidities (diabetes mellitus, arterial hypertension, dyslipidemia, chronic kidney disease, chronic lung disease, chronic liver disease, malignancy, and depression).

^b^Not included in the final multivariate analysis because of the lack of statistical significance.

^c^Hematological or solid organ malignancy.

**Figure 2:**
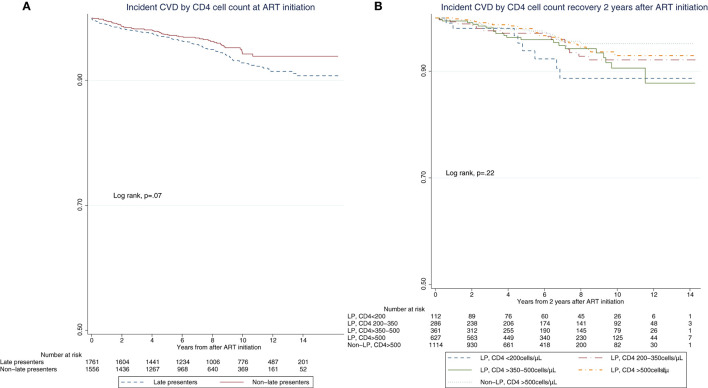
Kaplan–Meier curves of time to cardiovascular events. **(A)** Incident cardiovascular events by CD4 cell count at ART initiation. **(B)** Incident cardiovascular events by CD4 cell count 2 years after ART initiation.

Kaplan–Meier curves for incident CV events stratified by CD4 count at ART initiation (LP vs. non-LP) or by their 2-year CD4 recovery (non-LP with CD4 count >500 cells/μL *vs*. LP with CD4 count >500, >350–500, 200–350, and < 200 cells/μL) showed no significant differences between groups (log-rank test, *p* = 0.07 and *p* = 0.22, respectively) (Figures 2A, B).

In the univariate regression analysis, only LP with a baseline CD4 count of < 200 cells/μL had an increased risk of CV events compared to non-LP [IRR 1.71 (95%CI: 1.19–2.44)]. However, this association was no longer shown in the multivariate analysis adjusted for age, HIV transmission mode, calendar time, and comorbidities [aIRR 0.92 (95%CI: 0.62–1.36) and 0.84 (95%CI: 0.56–1.26) for LP with baseline CD4 count < 200 and 200–350 cells/μL, respectively] (Table 2). The risk of CV events increased significantly paralleling the age of PWH with no evidence of effect modification for LP (Supplementary Table 4). Other variables associated with increased risk were heterosexual males compared to MSM [aIRR 2.43 (95%CI: 1.62–3.67)], chronic liver disease [aIRR 2.38 (1.48–3.82)], and malignancy [aIRR 2.27 (1.20–4.30)]. Diabetes, arterial hypertension, dyslipidemia, chronic kidney or lung disease, and depression were not associated with an increased risk for CV events in the multivariate analysis.

### Mortality outcomes

Overall, 185 (5.6%) patients died during the study period (Supplementary Tables 5, 6). Among the 163 PWH experiencing a CV event, 31 died [26 LP and 5 non-LP, MR 40.4/1000PY (95%CI: 28.4–57.4)]. One-third of these deaths (*n* = 12, 38.7%) occurred within 3 months and 18 (58%) within the first year (Supplementary Figure 2). LP exhibited higher cumulative mortality (log-rank, *P* = 0.038) (Supplementary Figure 2); however, mortality was no longer higher in the multivariate analysis after adjusting for age, HIV transmission mode, and comorbidities [aMRR 1.24 (95%CI: 0.45–3.44)], although the absolute numbers were small. Women and individuals with chronic lung and/or liver disease had an increased mortality after a CV event [aMRR 5.89 (95%CI:1.35–25.60), 5.06 (95%CI:1.61–15.91) and 3.49 (95%CI:1.08–11.26), respectively] (Table 3).

**Table 3:** All-cause mortality in 163 people with HIV developing a first CV event.

	**Death (*n*)**	**MR per 1,000 PY**	**MRR (95% CI)**	**aMRR (95% CI)**
Total	31	40.4 (28.4–57.4)		
**Gender**				
Male	23	36.0 (23.9–54.2)	Ref (1)	
Female	8	62.1 (31.0–124.1)	1.72 (0.77–3.85)	
Age at baseline			1.06 (1.03–1.10)	1.07 (1.03–1.11)
**Route of HIV transmission**				
MSM	3	11.6 (3.7–35.9)	Ref (1)	Ref (1)
Heterosexual men	11	49.4 (27.4–89.2)	4.26 (1.19–15.28)	2.90 (0.76–11.03)
Women	5	74.5 (31.0–179.0)	6.43 (1.54–26.91)	5.89 (1.35–25.60)
IDU	8	57.3 (28.7–114.6)	4.95 (1.31–18.6)	2.58 (0.52–12.72)
Unknown	4	50.5 (19.0–134.5)	4.36 (0.98–19.48)	1.67 (0.30–9.19)
**Cardiovascular disease**				
Heart failure	9	42.0 (21.8–80.6)	2.13 (0.66–6.92)	
Ischemic heart disease	4	19.7 (7.4–52.4)	Ref (1)	
Cerebrovascular disease	14	56.2 (33.3–94.8)	2.85 (0.94–8.67)	
Peripheral vascular disease	4	39.8 (14.9–105.9)	2.02 (0.50–8.08)	
**Calendar time**				
2005–2009	1	120.8 (17.0–857.7)	3.45 (0.46–25.58)	
2010–2014	8	60.9 (30.4–121.7)	1.74 (0.77–3.90)	
2015–2021	22	35.0 (23.1–53.2)	Ref (1)	
**AIDS at baseline**				
Yes	11	89.5 (49.6–161.6)	2.88 (1.38–6.02)	
No	20	31.0 (2.0–48.1)	Ref (1)	
**CD4 cell count at ART initiation**				
Late presenters, CD4 ≤ 350 cells/μL	26	5.0 (34.0–73.4)	2.48 (0.95–6.45)	1.24 (0.45–3.44)
Non-late presenters, >350 cells/μL	5	20.2 (8.4–48.5)	Ref (1)	Ref (1)
**Comorbidity at ART initiation**				
**Diabetes mellitus**				
Yes	2	88.5 (22.1–354.0)	2.27 (0.54–9.53)	
No	29	38.9 (27.1–56.0)	Ref (1)	
**Dyslipidaemia**				
Yes	3	60.2 (19.4–186.8)	1.54 (0.47–5.08)	
No	28	39.0 (26.9–56.5)	Ref (1)	
**Arterial hypertension**				
Yes	3	41.5 (13.4–128.7)	1.03 (0.31–3.39)	
No	28	40.3 (27.8–58.3)	Ref (1)	
**Chronic kidney disease**				
Yes	1	107.9 (15.2–756.8)	2.73 (0.37–2.00)	
No	30	39.6 (27.7–56.6)	Ref (1)	
**Chronic lung disease**				
Yes	4	215.8 (81.0–575.0)	5.99 (2.10–17.11)	5.06 (1.61–15.91)
No	27	36.0 (24.7–52.6)	Ref (1)	Ref (1)
**Chronic liver disease**				
Yes	8	78.7 (39.3–157.3)	2.28 (1.02–5.09)	3.49 (1.08–11.26)
No	23	34.5 (23.0–52.0)	Ref (1)	Ref (1)
**Malignancy**				
Yes	3	77.4 (24.9–239.8)	2.01 (0.61–6.62)	
No	28	38.4 (26.5–55.6)	Ref (1)	
**Depression**				
Yes	2	40.4 (28.1–58.2)	0.99 (0.24–4.14)	
No	29	39.9 (1.0–159.7)	Ref (1)	

aMRR, adjusted mortality rate ratio; ART, antiretroviral therapy; IDU, injection drug use; MR, mortality rate; MRR, mortality rate ratio; MSM, men who have sex with men; PY, person-years.

### Sensitivity analysis

Given the known high morbidity and mortality due to AIDS-related conditions in LP during the first 2 years, we specifically analyzed the subset surviving the first 2 years after ART initiation (*n* = 2625 PWH) (Supplementary Figure 1). In this analysis, with 103 persons experiencing a CV event, we found similar results with no increased risk of CV events in LP compared to non-LP with 2-year CD4 count >500 cells/μL (reference population) after adjusting for age (time-updated), calendar time (time-updated), HIV transmission mode, and comorbidities, regardless of the 2-year CD4 count [aIRR 0.99 (0.43–2.26) and 0.82 (0.48–1.39), in LP with a 2-year CD4 count of < 200 cells/μL and 200–500 cells/μL, respectively] (Supplementary Tables 7, 8; Supplementary Figure 3).

## Discussion

In this multicenter, population-based, prospective cohort analysis, we observed that LP with no previous CV events at ART initiation did not have an increased long-term risk of CV events compared to non-LP. The definition included a composite endpoint of congestive heart failure, ischemic heart disease, cerebrovascular, and peripheral vascular disease. Mortality rates after CV events were high with ~40% of them occurring within the first 3 months after the event. Women and individuals with chronic lung and liver disease experienced particularly elevated mortality rates after CV events. Additional adjusted analyses including only PWH surviving the first 2 years after ART initiation yielded similar results, regardless of 2-year CD4 cell recovery, reinforcing the main findings of the study.

Several studies have shown that unremitting immune activation, persistent inflammation, and vascular dysfunction could be common in PWH and potentially associated with earlier atherosclerosis and premature CVD (25). LP have increased chronic inflammation due to persistent immune damage established before ART initiation (7), and higher levels of immune activation could theoretically play an additional role in the development of early CVD in this subpopulation. However, our results do not confirm an increased risk of CV events in LP compared to the rest of PWH. Although no differences were observed in the adjusted analysis, PWH with a CD4 count of < 200 cells/μL at ART initiation or after 2 years had an approximately unadjusted 2-fold increased risk of CV events compared to non-LP. This association is likely due to the confounding effect of age, calendar time, and various comorbidities, which themselves are independent risk factors for CVD. Not unexpectedly, an increased risk for CV events was seen in older PWH. Older people are at increased risk of both LP and CVD and in fact, the proportion of PWH older than 50 years is increasing, and about half of them have delayed presentation (4). On the other side, multimorbidity is common in LP, and a recent cross-sectional Italian study showed that multimorbidity was more common in LP compared to non-LP across all age distributions (26). All this suggests that data suggesting increased rates of CV events in LP could be biased by unadjusted confounders.

Calendar time could theoretically play a role as well, with lower CV risk in recent years, paralleling with improved cardiovascular risk management in PWH and increased safety and efficacy of ART, although this trend was not confirmed in our cohort. Recent investigations on the risk of CVD using INSTI-based regimens, the currently recommended first-line choices, have yielded inconsistent results. A study from the RESPOND cohort consortium, which included 29340 individuals, 14000 of whom were given an INSTI, have reported an increased risk of CVD in the first 2 years after INSTI initiation compared to initiating other regimens (27). However, another recent study in the Swiss HIV Cohort, which included 5362 individuals initiating ART after May 2008, 1837 individuals initiated an INSTI-based regimen, found no such risk (28). In the following years, it will be important to determine whether the link between INSTI usage and CVD is due to INSTI use or to unmeasured confounding. On the other side, the use of INSTI has been recently been associated with several studies with a more favorable immune recovery (29, 30).

Our results are in concordance with the results from the Spanish CoRIS cohort including patients initiating ART between 2004 and 2018, where they found no evidence of an increment of CV events in LP compared to non-LP. However, the analysis was not adjusted for previous comorbidities that could play a major role in the development of CVD (14). Similarly, in a study from the Veterans Aging Cohort including individuals from 1996 to 2012, no increased risk of CVD was seen with individuals initiating ART with a CD4 count of < 200 cells/μL compared to those ≥200 cells/μL (15). In a Dutch Athena Cohort study including patients between 1998 and 2009, no association was found between LP with a 2-year CD4 count of < 200 cells/μL and risk of CVD in the adjusted analysis, although individuals with previous CVD were not excluded (13).

In contrast, other studies have described an association between CD4 cell count below 200 cells/μL and a higher risk of CVD. Two cohort studies from the US (Veterans Aging Cohort and North American AIDS Cohort Collaboration on Research and Design [NA-ACCORD]) and two metanalyses, all including participants before the year 2013, have shown that CD4 counts < 200 cells/μL were associated with increased CV risk, including incident myocardial infarction (aHR 2.02; 95% CI, 1.42–2.88), ischemic stroke (aHR 1.66; 95% CI, 1.30–2.12), and peripheral artery disease (HR, 1.91; 95% CI, 1.71–2.13) (16–20). However, most of these studies included immunological non-responders, who fail to reconstitute their CD4 cell count despite virological suppression on ART, who are known to have increased long-term morbidity and mortality risk (31). Furthermore, a significant proportion of individuals in these studies had unsuppressed viremia at the moment of the event, either because they were not on ART at the time or because they belonged to an HIV subpopulation that was heavily treated or had poor treatment adherence. HIV unsuppressed viremia is strongly correlated with chronic inflammation, ongoing immune activation, and accelerated atherosclerosis and could have confounded the finding. Our study included very few immunological non-responders with a 2-year CD4 count of < 200 cells/μL and CV events to draw reliable conclusions in this subpopulation. These patients had higher rates of detectable viremia, comorbidities (AIDS-defining events, chronic liver disease, and malignancies), and lower educational levels, which could also influence their CV risk. However, in the whole cohort, only 6.4% of LP had detectable viremia 2 years after ART initiation.

We also identified an increased risk of CV events in patients with chronic liver disease and malignancies. This association must be interpreted with caution given that other risk factors, such as tobacco and alcohol abuse, could be possible underlying confounders in these groups. A higher frequency of smoking, alcohol, and recreational drug abuse has been, indeed, associated with higher rates of CVD (32).

Mortality after a CV event was very high. However, we did not observe mortality differences between LP and non-LP. Nevertheless, the absolute numbers were low and do not allow for an in-depth analysis to establish robust conclusions.

To our knowledge, this is the first assessment of CV event rates in LP starting ART in a more recent calendar period (2005–2019), including integrase inhibitor use, with information on important comorbidities related to CVD enabling the performance of an accurate adjusted analysis. We adjusted for calendar time because both the management and prognosis of CVD and ART initiation guidelines have varied over time. Other strengths of this study include the prospective, population-based, multicenter HIV-cohort design, including 84% of all PWH followed in Catalonia and the long observation period. We had access to high-quality, real-world health data generated by the public health system of Catalonia, including all hospital and primary care diagnoses, thus including accurate information on CV events, comorbidities, and mortality. We assessed the risk of CVD in PWH initiating ART and in PWH surviving the first 2 years, to avoid the vulnerable first 2-year period after HIV diagnosis with high risk for severe comorbidities and complications.

In terms of limitations, we did not have access to causes of death, neither overall nor in CV event-related deaths, and a low prevalence of the latter limits a more thorough interpretation. Furthermore, the percentage of subjects with a CD4 count of < 200 cells/μL after 2 years of ART initiation was low (only 8%) and it is therefore not possible to draw definitive conclusions to establish a relationship between immunological non-response and CVD. We had no information on current smoking, alcohol consumption, or substance abuse other than injectable drug usage, all of which are known independent risk factors for CVD. We included chronic lung and liver disease in the model, which could be interpreted as a surrogate marker, especially for the first two risk factors. Finally, the number of events was low, and type II errors in this analysis cannot be ruled out.

In conclusion, CVD remains a common cause of morbidity and mortality among PWH. LP with no prior CV events at ART initiation did not have an increased long-term risk of CV events compared with non-LP. Our results call for risk stratification and identification of PWH at increased risk of future CVD based on traditional risk factors and comorbidities linked to increased CV risk, to focus risk-reducing strategies in these subjects. When initiating successful ART, late HIV presentation does not increase by itself the risk for CVD.

## Data availability statement

The data analyzed in this study is subject to the following licenses/restrictions: The data collected for this study are available from the Centre for Epidemiological Studies of Sexually Transmitted Diseases and HIV/AIDS in Catalonia (CEEISCAT), the coordinating center of the PISCIS Cohort Study and from each of the collaborating hospitals upon request. Requests can be made via https://pisciscohort.org/contacte/. The study protocol, the statistical codebook and codes for the analysis can be requested from RM-I (raquel@bisaurin.org).

## Ethics statement

The studies involving human participants were reviewed and approved by Germans Trias i Pujol University Hospital's Clinical Research Ethics Committee, Reference Number EO-11-108. Written informed consent for participation was not required for this study in accordance with the national legislation and the institutional requirements.

## Author contributions

RM-I conducted the research and analyzed the data. RM-I, MV-F, and JL wrote the first draft of the manuscript. All authors contributed to the interpretation of the results and revision of the manuscript and gave their final approval to the manuscript.

## PISCIS study group

The PISCIS study group includes: Jordi Casabona, A. Esteve, Andreu Bruguera, Sergio Moreno-Fornés (CEEISCAT), Jose M. Miró, J. Mallolas, E. Martínez, JL Blanco, M. Laguno, M. Martínez-Rebollar, B. Torres, A. Gonzalez-Cordon (Hospital Clínic-Idibaps, Universitat de Barcelona), Elisa De Lazzari (Hospital Clínic-Idibaps), Arkaitz Imaz (Unitat de VIH i ITS, Servei de Malalties Infeccioses, Hospital Universitari de Bellvitge, IDIBELL), Pere Domingo (Unitat de VIH/SIDA Hospital de la Santa Creu i Sant Pau), Josep María Llibre (Fundació Lluita contra la Sida-Hospital Universitari Germans Trias i Pujol-Universitat Autònoma de Barcelona), Francisco Fanjul (Servei Medicina Interna, Hospital Universitari Son Espases), Gemma Navarro (Unitat de VIH/SIDA, Parc Tauli Hospital Universitari-Universitat Autònoma de Barcelona), Vicenç Falcó Ferrer [Servei de Malalties Infeccioses, Hospital Universitari Vall d'Hebron, Vall d'Hebron Research Institute (VHIR)], and Hernando Knobel (Servei de Malalties Infeccioses, Hospital del Mar).
