# Supplementary material for: Cardiovascular events in delayed presentation of HIV: the prospective PISCIS cohort study
Source: Front Med (Lausanne). 2023 Jun 21;10:1182359. doi: 10.3389/fmed.2023.1182359 (PMC10321350; doi:10.3389/fmed.2023.1182359)
Supplement: Supplementary file 1 [file Data_Sheet_1.docx]

**Supplementary data**

**Testing model assumptions**

**Poisson regression assumptions:**

- Response variable is count data (0 or 1).

- Explanatory variables are continuous, dichotomous, or ordinal.

- Observations are independent.

- Event rate is constant within each interval of follow-up.

-Counts follow a Poisson distribution. We check for equi-dispersion: the mean value of data equals the variance value (mean=0.0392381 and variance=0.0377128).

We performed both Poisson and Exponential-Poisson regression. When testing the model fitness, the Exponential-Poisson regression, with parametric survival-time model (STATA command *streg*), had a better model fitness than a simple Poisson model (AIC= 919 in exponential- Poisson regression vs AIC=1180 Poisson regression). For this reason, we chose to use Exponential-Poisson regression.

**eTable 1. Description of ICD-10 codes used to define the comorbidities assess in the study (based on the Swedish National Study of Aging and Care in Kungsholmen (SNAC-K) coding).**

| **ISCHEMIC HEART DISEASE** | |
| --- | --- |
| **Included ICD-10 codes and labels** | |
| I20 | Angina pectoris |
| I21 | Acute myocardial infarction |
| I22 | Subsequent myocardial infarction |
| I24 | Other acute ischaemic heart diseases |
| I25 | Chronic ischaemic heart disease |
| Z951 | Presence of aortocoronary bypass graft |
| Z955 | Presence of coronary angioplasty implant and graft |
| Additional parameters | Use of organic nitrates (C01DA) or ranolazine (C01EB18) |
| **HEART FAILURE** | |
| **Included ICD-10 codes and labels** | |
| I110 | Hypertensive heart disease with (congestive) heart failure |
| I130 | Hypertensive heart and renal disease with (congestive) heart failure |
| I132 | Hypertensive heart and renal disease with both (congestive) heart failure and renal failure |
| I27 | Other pulmonary heart diseases |
| I280 | Arteriovenous fistula of pulmonary vessels |
| I42 | Cardiomyopathy |
| I43 | Cardiomyopathy in diseases classified elsewhere |
| I50 | Heart failure |
| I515 | Myocardial degeneration |
| I517 | Cardiomegaly |
| I528 | Other heart disorders in other diseases classified elsewhere |
| Z941 | Heart transplant status |
| Z943 | Heart and lungs transplant status |
| **CEREBROVASCULAR DISEASE** | |
| **Included ICD-10 codes and labels** | |
| G45 | Transient cerebral ischaemic attacks and related syndromes |
| G46 | Vascular syndromes of brain in cerebrovascular diseases |
| I60 | Subarachnoid haemorrhage |
| I61 | Intracerebral haemorrhage |
| I62 | Other nontraumatic intracranial haemorrhage |
| I63 | Cerebral infarction |
| I64 | Stroke, not specified as haemorrhage or infarction |
| I67 | Other cerebrovascular diseases |
| I69 | Sequelae of cerebrovascular disease |
| **PERIPHERAL VASCULAR DISEASE** | |
| **Included ICD-10 codes and labels** | |
| I702 | Atherosclerosis of arteries of extremities |
| I73 | Other peripheral vascular diseases |
| I792 | Peripheral angiopathy in diseases classified elsewhere |
| I798 | Other disorders of arteries, arterioles and capillaries in diseases classified elsewhere |
| Additional parameters | Use of cilostazol (B01AC23) |
| **Excluded ICD-10 codes and labels** | |
| I731 | Thromboangiitis obliterans [Buerger] |
| I738 | Other specified peripheral vascular diseases |
| **CHRONIC KIDNEY DISEASES** | |
| **Included ICD-10 codes and labels** | |
| I120 | Hypertensive renal disease with renal failure |
| I130 | Hypertensive heart and renal disease with (congestive) heart failure |
| I131 | Hypertensive heart and renal disease with renal failure |
| I132 | Hypertensive heart and renal disease with both (congestive) heart failure and renal failure |
| I139 | Hypertensive heart and renal disease, unspecified |
| N01 | Rapidly progressive nephritic syndrome |
| N03 | Chronic nephritic syndrome |
| N04 | Nephrotic syndrome |
| N05 | Unspecified nephritic syndrome |
| N07 | Hereditary nephropathy, not elsewhere classified |
| N08 | Glomerular disorders in diseases classified elsewhere |
| N11 | Chronic tubulo-interstitial nephritis |
| N183 | Chronic kidney disease, stage 3 |
| N184 | Chronic kidney disease, stage 4 |
| N185 | Chronic kidney disease, stage 5 |
| N189 | Chronic kidney disease, unspecified |
| Q60 | Renal agenesis and other reduction defects of kidney |
| Q611 | Polycystic kidney, autosomal recessive |
| Q612 | Polycystic kidney, autosomal dominant |
| Q613 | Polycystic kidney, unspecified |
| Q614 | Renal dysplasia |
| Q615 | Medullary cystic kidney |
| Q618 | Other cystic kidney diseases |
| Q619 | Cystic kidney disease, unspecified |
| Z905 | Acquired absence of kidney |
| Z940 | Kidney transplant status |
| Additional parameters | Glomerular filtration rate <60 ml/min/1.73m2 (assessed using the CKD-EPI equation)^2^ |
| **CHRONIC LIVER DISEASES** | |
| **Included ICD-10 codes and labels** | |
| B18 | Chronic viral hepatitis |
| K70 | Alcoholic liver disease |
| K713 | Toxic liver disease with chronic persistent hepatitis |
| K714 | Toxic liver disease with chronic lobular hepatitis |
| K715 | Toxic liver disease with chronic active hepatitis |
| K717 | Toxic liver disease with fibrosis and cirrhosis of liver |
| K721 | Chronic hepatic failure |
| K73 | Chronic hepatitis, not elsewhere classified |
| K74 | Fibrosis and cirrhosis of liver |
| K753 | Granulomatous hepatitis, not elsewhere classified |
| K754 | Autoimmune hepatitis |
| K758 | Other specified inflammatory liver diseases |
| K761 | Chronic passive congestion of liver |
| K766 | Portal hypertension |
| K767 | Hepatorenal syndrome |
| K778 | Liver disorders in other diseases classified elsewhere |
| Q446 | Cystic disease of liver |
| Z944 | Liver transplant status |
| **Excluded ICD-10 codes and labels** | |
| K700 | Alcoholic fatty liver |
| K701 | Alcoholic hepatitis |
| **COPD, EMPHYSEMA, CHRONIC BRONCHITIS** | |
| **Included ICD-10 codes and labels** | |
| J41 | Simple and mucopurulent chronic bronchitis |
| J42 | Unspecified chronic bronchitis |
| J43 | Emphysema |
| J44 | Other chronic obstructive pulmonary disease |
| J47 | Bronchiectasis |
| Additional parameters | Use of anticholinergics (R03BB) |
| **DEMENTIA** | |
| **Included ICD-10 codes and labels** | |
| F00 | Dementia in Alzheimer disease |
| F01 | Vascular dementia |
| F02 | Dementia in other diseases classified elsewhere |
| F03 | Unspecified dementia |
| F051 | Delirium superimposed on dementia |
| G30 | Alzheimer disease |
| G31 | Other degenerative diseases of nervous system, not elsewhere classified |
| Additional parameters | Diagnostic and Statistical Manual of Mental Disorders, Third Edition, Revised (assessed by two different physicians, and a third one in case of disagreement)  Use of anticholinesterases (N06DA) or memantine (N06DX01) |
| **DEPRESSION AND MOOD DISEASES** | |
| **Included ICD-10 codes and labels** | |
| F30 | Manic episode |
| F31 | Bipolar affective disorder |
| F32 | Depressive episode |
| F33 | Recurrent depressive disorder |
| F34 | Persistent mood [affective] disorders |
| F38 | Other mood [affective] disorders |
| F39 | Unspecified mood [affective] disorder |
| F412 | Mixed anxiety and depressive disorder |
| **DIABETES** | |
| **Included ICD-10 codes and labels** | |
| E10 | Insulin-dependent diabetes mellitus |
| E11 | Non-insulin-dependent diabetes mellitus |
| E13 | Other specified diabetes mellitus |
| E14 | Unspecified diabetes mellitus |
| E891 | Postprocedural hypoinsulinaemia |
| Additional parameters | Glycated hemoglobin (A1C) ≥6.5%  Use of antidiabetics (A10) |
| **DYSLIPIDEMIA** | |
| **Included ICD-10 codes and labels** | |
| E78 | Disorders of lipoprotein metabolism and other lipidaemias |
| **HEMATOLOGICAL NEOPLASMS** | |
| **Included ICD-10 codes and labels** | |
| C81 | Hodgkin lymphoma |
| C82 | Follicular lymphoma |
| C83 | Non-follicular lymphoma |
| C84 | Mature T/NK-cell lymphomas |
| C85 | Other and unspecified types of non-Hodgkin lymphoma |
| C86 | Other specified types of T/NK-cell lymphoma |
| C88 | Malignant immunoproliferative diseases |
| C90 | Multiple myeloma and malignant plasma cell neoplasms |
| C91 | Lymphoid leukaemia |
| C92 | Myeloid leukaemia |
| C93 | Monocytic leukaemia |
| C94 | Other leukaemias of specified cell type |
| C95 | Leukaemia of unspecified cell type |
| C96 | Other and unspecified malignant neoplasms of lymphoid, haematopoietic and related tissue |
| **HYPERTENSION** | |
| **Included ICD-10 codes and labels** | |
| I10 | Essential (primary) hypertension |
| I11 | Hypertensive heart disease |
| I12 | Hypertensive renal disease |
| I13 | Hypertensive heart and renal disease |
| I15 | Secondary hypertension |
| Additional parameters | Blood pressure ≥140/90 mmHg |
| **SOLID NEOPLASMS** | |
| **Included ICD-10 codes and labels** | |
| C | Malignant neoplasms |
| D00 | Carcinoma in situ of oral cavity, oesophagus and stomach |
| D01 | Carcinoma in situ of other and unspecified digestive organs |
| D02 | Carcinoma in situ of middle ear and respiratory system |
| D03 | Melanoma in situ |
| D04 | Carcinoma in situ of skin |
| D05 | Carcinoma in situ of breast |
| D06 | Carcinoma in situ of cervix uteri |
| D07 | Carcinoma in situ of other and unspecified genital organs |
| D09 | Carcinoma in situ of other and unspecified sites |
| D320 | Benign neoplasm: Cerebral meninges |
| D321 | Benign neoplasm: Spinal meninges |
| D329 | Benign neoplasm: Meninges, unspecified |
| D330 | Benign neoplasm: Brain, supratentorial |
| D331 | Benign neoplasm: Brain, infratentorial |
| D332 | Benign neoplasm: Brain, unspecified |
| D333 | Benign neoplasm: Cranial nerves |
| D334 | Benign neoplasm: Spinal cord |
| Q85 | Phakomatoses, not elsewhere classified |
| **Excluded ICD-10 codes and labels** | |
| C81 | Hodgkin lymphoma |
| C82 | Follicular lymphoma |
| C83 | Non-follicular lymphoma |
| C84 | Mature T/NK-cell lymphomas |
| C85 | Other and unspecified types of non-Hodgkin lymphoma |
| C86 | Other specified types of T/NK-cell lymphoma |
| C88 | Malignant immunoproliferative diseases |
| C90 | Multiple myeloma and malignant plasma cell neoplasms |
| C91 | Lymphoid leukaemia |
| C92 | Myeloid leukaemia |
| C93 | Monocytic leukaemia |
| C94 | Other leukaemias of specified cell type |
| C95 | Leukaemia of unspecified cell type |
| C96 | Other and unspecified malignant neoplasms of lymphoid, haematopoietic and related tissue |

**eTable 2. Additional clinical and drug-related parameters used in SNAC-K to define specific chronic conditions.**

| **Condition** | **Clinical and drug-related parameters** |
| --- | --- |
| **Chronic kidney diseases** | Glomerular filtration rate <60 ml/min/1.73m2 (assessed using the CKD-EPI equation)^1^ |
| **COPD, Emphysema, Chronic Bronchitis** | Use of anticholinergics (R03BB) |
| **Dementia** | Diagnostic and Statistical Manual of Mental Disorders, Third Edition, Revised^2^ (assessed by two different physicians, and a third one in case of disagreement)  Use of anticholinesterases (N06DA) or memantine (N06DX01) |
| **Diabetes** | Glycated hemoglobin (A1C) ≥6.5%^3^  Use of antidiabetics (A10) |
| **Hypercholesterolemia** | Serum total cholesterol ≥6.22 mmol/L^4^ |
| **Hypertension** | Blood pressure ≥140/90 mmHg^5^ |
| **Ischemic heart disease** | Use of organic nitrates (C01DA) or ranolazine (C01EB18) |
| **Peripheral vascular disease** | Use of cilostazol (B01AC23) |

^*^The ATC codes corresponding to each drug are shown in brackets. Only those drugs that can be unequivocally linked to chronic conditions were considered. The selection of ATC codes was based on a literature review and the clinical judgment of physicians.

NOTE: The criteria presented in this table were used in addition to the diagnoses assigned in SNAC-K.

^1^ Levey AS, Stevens LA, Schmid CH, Zhang YL, Castro AF, Feldman HI, et al. A new equation to estimate glomerular filtration rate. Ann Intern Med. 2009;150(9):604–12.

^2^ American Psychiatric Association. Diagnostic and statistical manual of mental disorders (3rd ed., text rev.). Washington, DC; 1987.

^3^ American Diabetes Association. Standards of Medical Care in Diabetes. Diabetes Care. 2016;39(Suppl 1):S4-5.

^4^ European Association for Cardiovascular Prevention & Rehabilitation, Reiner Z, Catapano AL, De Backer G, Graham I, Taskinen MR, et al. ESC/EAS Guidelines for the management of dyslipidaemias: the Task Force for the management of dyslipidaemias of the European Society of Cardiology (ESC) and the European Atherosclerosis Society (EAS). Eur Heart J. 2011;32(14):1769–818.

^5^ Mancia G, De Backer G, Dominiczak A, Cifkova R, Fagard R, Germano G, et al. 2007 Guidelines for the management of arterial hypertension: The Task Force for the Management of Arterial Hypertension of the European Society of Hypertension (ESH) and of the European Society of Cardiology (ESC). Eur Heart J. 2007;28(12):1462–536.

**eFigure 1. Flowchart of cohort construction. Including all PLWH initiating ART in the study period, surviving the first two years with the availability of CD4 cell count at 2 years after ART initiation.**


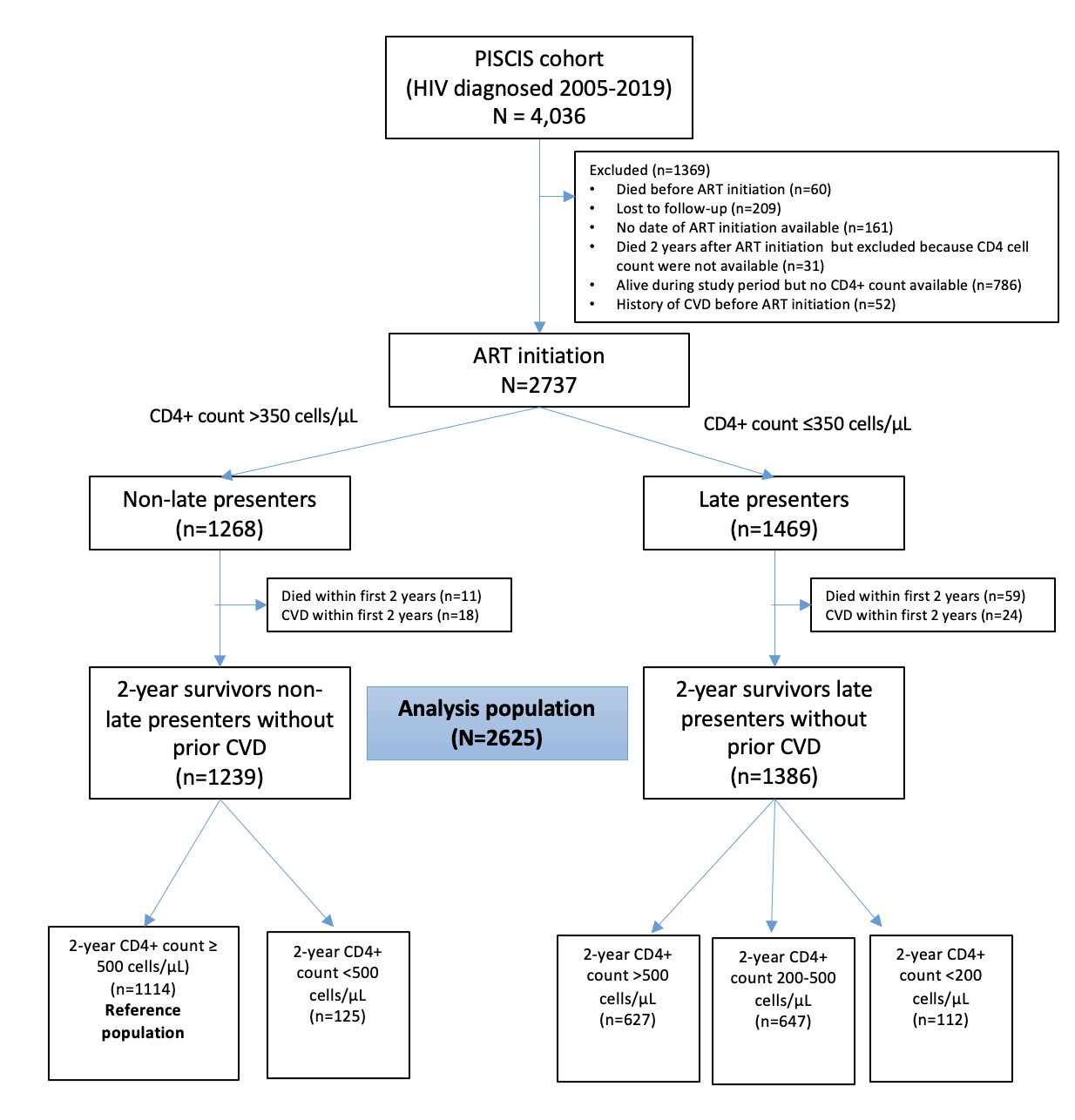


**eTable 3. Incidence rates of the different cardiovascular events.**

|  | Events  N (%) | Person-years at risk | IR per 1000 PYR (95% CI) | IRR in late presenters vs non-late presenters | aIRR in late presenters vs non-late presenters^1^ |
| --- | --- | --- | --- | --- | --- |
| Incident first CVE | 163 (4.9) | 26589.1 | 6.1 (5.3-7.1) | 1.34 (0.98-1.86) | 1.09 (0.79-1.51) |
| Heart failure | 48 (29.4) | 27137.4 | 1.8 (1.3-2.3) | 0.80 (0.45-1.41) | 0.68 (0.38-1.20) |
| Ischemic heart disease | 42 (25.8) | 27153.5 | 1.5 (1.1-2.1) | 1.48 (0.78-2.81) | 1.16 (0.60-2.21) |
| Cerebrovascular disease | 53 (32.5) | 27107.4 | 2.0 (1.5-2.6) | 1.71 (0.95-3.08) | 1.37 (0.76-2.48) |
| Peripheral vascular disease | 20 (12.3) | 27249.8 | 0.7 (0.5-1.1) | 2.21 (0.81-6.10) | 1.45 (0.52-3.99) |

Abbreviations: aIRR, adjusted incidence rate ratio; CVE, cardiovascular event; IR, incidence rate; IRR, incidence rate ratio.

^1^ Adjusted for age.

**eTable 4. Incidence rate ratios of first CV event in LP vs non-LP surviving the first two years according to different baseline characteristics.**

|  | IRR (95% CI) |
| --- | --- |
| Total | 1.35 (0.98-1.86) |
| Gender |  |
| Male | 1.40 (0.98-1.98) |
| Age (time-updated) (years)^1^ |  |
| <40 | 0.63 (0.30-1.34) |
| 40-49 | 1.10 (0.64-1.91) |
| >50 | 1.33 (0.80-2.20) |
| Transmission mode |  |
| MSM | 0.92 (0.55-1.55) |
| Heterosexual men | 1.18 (0.60-2.32) |
| Women | 0.72 (0.27-1.94) |
| IDU | 1.15 (0.49-2.72) |
| Unknown/other | 2.26 (0.64-8.00) |
| HIV viral load >200 c/ml 2 years after ART initiation |  |
| Yes | 1.59 (0.49-5.15) |
| No | 1.24 (0.86-1.80) |
| Calendar time (time-updated) |  |
| 2005-2009 | 1.97 (0.24-15.05) |
| 2010-2014 | 1.49 (0.79-2.83) |
| 2015-2021 | 1.32 (0.90-1.94) |
| Comorbidities at baseline |  |
| Diabetes | 0.86 (0.17-4.27) |
| Arterial hypertension | 1.16 (0.44-3.10) |
| Dyslipidaemia | 0.74 (0.24-2.25) |
| Chronic kidney disease | - |
| Chronic lung disease | - |
| Chronic liver disease | 0.96 (0.42-2.17) |
| Malignancy^2^ | 0.46 (0.14-1.51) |
| Depression | 0.93 (0.33-2.64) |

^1^ Test for interaction p=0.34.

^2^ Haematological or solid organ malignancy.

**eTable 5. Overall mortality in the study period in PLWH initiating ART (n=3317).**

|  | Deaths in the study period | Person-years  (PY) | MR per 1000 PY (95%CI) | MRR (95%CI) | aMRR^1^ (95%CI) |
| --- | --- | --- | --- | --- | --- |
| Total | 185 | 27356.7 | 6.8 (5.9-7.8) |  |  |
| Gender |  |  |  |  |  |
| Male | 158 | 23102.0 | 6.8 (5.8-8.0) | Ref (1) |  |
| Female | 27 | 4247.9 | 6.4 (4.4-9.3) | 0.93 (0.62-1.40) |  |
| Age (continuous) |  |  |  | 1.08 (1.06-1.09) | 1.06 (1.04-1.07) |
| CD4 cell count at ART initiation |  |  |  |  |  |
| Late presenters, CD4 ≤350 cells/µL | 150 | 15751.2 | 9.5 (8.1-11.2) | 3.16 (2.19-4.56) | 1.92 (1.31-2.80) |
| Non-late, CD4 >350 cells/µL | 35 | 11605.5 | 3.0 (2.2-4.2) | Ref (1) | Ref (1) |
| HIV transmission mode |  |  |  |  |  |
| MSM | 51 | 16147.5 | 3.2 (2.4-4.2) | Ref (1) | Ref (1) |
| Heterosexual men | 50 | 3984.6 | 12.5 (9.5-16.6) | 3.97 (2.69-5.87) | 2.14 (1.42-3.24) |
| Women | 16 | 3443.8 | 4.6 (2.8-7.6) | 1.47 (0.84-2.58) | 1.15 (0.66-2.03) |
| IDU | 51 | 2180.6 | 23.4 (17.8-30.8) | 7.41 (5.02-10.92) | 3.95 (2.57-6.08) |
| Unknown | 17 | 1600.3 | 10.6 (6.6-17.1) | 3.36 (1.94-5.82) | 1.98 (1.13-3.47) |
| Calendar time |  |  |  |  |  |
| 2015-2021 | 99 | 18014.8 | 5.5 (4.5-6.7) | Ref (1) | Ref (1) |
| 2010-2014 | 51 | 7554.2 | 6.8 (5.1-8.9) | 1.23 (0.88-1.72) | 1.08 (0.77-1.51) |
| 2005-2009 | 35 | 1787.7 | 19.6 (14.1-27.3) | 3.56 (2.42-5.24) | 2.45 (1.65-3.64) |
| AIDS-defining event at ART initiation |  |  |  |  |  |
| Yes | 44 | 2223.9 | 19.8 (14.7-26.6) | 3.53 (2.51-4.95) | 1.50 (1.04-2.16) |
| No | 141 | 25132.8 | 5.6 (4.8-6.6) | Ref (1) | Ref (1) |
| Comorbidity at ART initiation |  |  |  |  |  |
| Diabetes mellitus |  |  |  |  |  |
| Yes | 5 | 210.0 | 23.8 (9.9-57.2) | 3.59 (1.48-8.73) | 0.82 (0.32-2.10) |
| No | 180 | 27146.7 | 6.6 (5.7-7.7) | Ref (1) | Ref (1) |
| Arterial hypertension |  |  |  |  |  |
| Yes | 10 | 1190.9 | 8.4 (4.5-15.6) | 1.26 (0.66-2.37) |  |
| No | 175 | 26165.8 | 6.7 (5.8-7.8) | Ref (1) |  |
| Dyslipidaemia |  |  |  |  |  |
| Yes | 4 | 836.3 | 4.8 (1.8-12.7) | 0.70 (0.26-1.89) |  |
| No | 181 | 26520.4 | 6.8 (5.9-7.9) | Ref (1) |  |
| Chronic kidney disease |  |  |  |  |  |
| Yes | 2 | 106.0 | 18.9 (4.7-75.4) | 2.81 (0.70-11.31) |  |
| No | 183 | 18859.9 | 6.7 (5.8-7.8) | Ref (1) |  |
| Chronic lung disease |  |  |  |  |  |
| Yes | 8 | 244.9 | 32.7 (16.3-65.3) | 5.00 (2.46-10.16) | 1.44 (0.68-3.07) |
| No | 177 | 27111.8 | 6.5 (5.6-7.6) | Ref (1) | Ref (1) |
| Chronic liver disease |  |  |  |  |  |
| Yes | 31 | 1331.2 | 23.3 (16.4-33.1) | 3.94 (2.68-5.79) | 1.98 (1.29-3.05) |
| No | 154 | 26025.5 | 5.9 (5.1-6.9) | Ref (1) | Ref (1) |
| Malignancy^2^ |  |  |  |  |  |
| Yes | 21 | 5292 | 39.7 (25.9-60.9) | 6.49 (4.12-10.22) | 3.90 (2.40-6.33) |
| No | 164 | 26827.4 | 6.1 (5.2-7.1) | Ref (1) |  |

Abbreviations: aMRR, adjusted mortality rate ratio; ART, antiretroviral therapy; IDU, injection drug use; MR, mortality rate; MRR, mortality rate ratio; MSM, men who have sex with men.

^1^ The aMRR was adjusted for age, transmission mode, CD4 cell count at ART initiation, diabetes mellitus, chronic lung disease, chronic liver disease, malignancy, and calendar time and AIDS-defining event.

^2^ Haematological or solid organ malignancy.

**eTable 6. Overall mortality in the study period in PLWH surviving the first two years and with available CD4 cell count two years after ART initiation (n=2625).**

|  | Deaths in the study period | Person-years  (PY) | MR per 1000 PY (95%CI) | MRR (95%CI) | aMRR^1^ (95%CI) |
| --- | --- | --- | --- | --- | --- |
| Total | 99 | 18277.2 | 5.4 (4.4-6.6) |  |  |
| Gender |  |  |  |  |  |
| Male | 88 | 15454.8 | 5.7 (4.6-7.0) | Ref (1) |  |
| Female | 11 | 2822.4 | 3.9 (2.2-7.0) | 0.68 (0.37-1.28) |  |
| Age (continuous) |  |  |  | 1.09 (1.07-1.11) | 1.07 (1.05-1.09) |
| CD4 cell count at ART initiation |  |  |  |  |  |
| Late presenters, CD4 ≤350 cells/µL | 79 | 10839.7 | 7.3 (5.8-9.1) | 2.71 (1.66-4.43) |  |
| Non-late, CD4 >350 cells/µL | 20 | 7437.5 | 2.7 (1.7-4.2) | Ref (1) |  |
| CD4 cell count 2 years after ART initiation |  |  |  |  |  |
| Late presenters, CD4 <200 cells/µL | 21 | 864.0 | 24.3 (15.8-37.3) | 9.92 (5.18-19.02) | 4.21 (2.05-8.65) |
| Late presenters, CD4 200-500 cells/µL | 44 | 5144.6 | 8.6 (6.4-11.5) | 3.49 (1.97-6.19) | 1.81 (0.99-3.33) |
| Late presenters, CD4 >500 cells/µL | 14 | 4831.2 | 2.9 (1.7-4.9) | 1.18 (0.58-2.42) | 0.94 (0.45-1.95) |
| Non-late presenters, CD4 ≤500 cells/µL | 4 | 904.8 | 4.4 (1.7-11.8) | 1.81 (0.60-5.40) | 1.44 (0.48-4.36) |
| Non-late presenters, CD4 >500 cells/µL | 16 | 6532.7 | 2.4 (1.5-4.0) | Ref (1) | Ref (1) |
| HIV transmission mode |  |  |  |  |  |
| MSM | 28 | 10901.3 | 2.6 (1.8-3.7) | Ref (1) | Ref (1) |
| Heterosexual men | 33 | 2658.9 | 12.4 (8.8-17.5) | 4.83 (2.92-8.00) | 2.31 (1.34-3.98) |
| Women | 9 | 2309.7 | 3.9 (2.0-7.5) | 1.52 (0.72-3.21) | 1.12 (0.52-2.40) |
| IDU | 21 | 1373.4 | 15.3 (10.0-23.5) | 5.95 (3.38-10.48) | 2.06 (1.06-3.99) |
| Unknown | 8 | 1033.8 | 7.7 (3.9-15.5) | 3.01 (1.37-6.61) | 1.48 (0.66-3.33) |
| Calendar time |  |  |  |  |  |
| 2015-2021 | 68 | 13549.5 | 5.0 (4.0-6.4) | Ref (1) | Ref (1) |
| 2010-2014 | 25 | 4300.6 | 5.8 (3.9-8.6) | 1.16 (0.73-1.83) | 0.94 (0.59-1.50) |
| 2005-2009 | 6 | 427.1 | 14.0 (6.3-31.3) | 2.80 (1.21-6.45) | 1.84 (0.79-4.30) |
| AIDS before 2 years after ART initiation |  |  |  |  |  |
| Yes | 23 | 1685.7 | 13.6 (9.1-20.5) | 2.98 (1.87-4.75) | 1.16 (0.69-1.93) |
| No | 76 | 16591.5 | 4.6 (3.7-5.7) | Ref (1) |  |
| Comorbidity at 2 years after ART initiation |  |  |  |  |  |
| Diabetes mellitus |  |  |  |  |  |
| Yes | 6 | 246.3 | 24.4 (10.9-54.2) | 4.72 (2.07-10.78) | 1.38 (0.57-3.36) |
| No | 93 | 18030.9 | 5.2 (4.2-6.3) | Ref (1) | Ref (1) |
| Arterial hypertension |  |  |  |  |  |
| Yes | 11 | 1240.1 | 8.9 (4.9-16.0) | 1.72 (0.92-3.21) |  |
| No | 88 | 17037.1 | 5.2 (4.2-6.4) | Ref (1) |  |
| Dyslipidaemia |  |  |  |  |  |
| Yes | 3 | 937.2 | 3.2 (1.0-9.9) | 0.58 (0.18-1.82) |  |
| No | 96 | 17340.0 | 5.5 (4.5-6.8) | Ref (1) |  |
| Chronic kidney disease |  |  |  |  |  |
| Yes | - | 107.3 | - | - |  |
| No | 99 | 18169.9 | 5.4 (4.5-6.6) |  |  |
| Chronic lung disease |  |  |  |  |  |
| Yes | 6 | 234.2 | 25.6 (11.5-57.0) | 4.97 (2.18-11.35) | 1.38 (0.56-3.42) |
| No | 93 | 18043.0 | 5.2 (4.2-6.3) | Ref (1) | Ref (1) |
| Chronic liver disease |  |  |  |  |  |
| Yes | 25 | 1435.7 | 17.4 (11.8-25.8) | 3.96 (2.52-6.24) | 2.34 (1.38-3.97) |
| No | 74 | 16841.5 | 4.4 (3.5-5.5) | Ref (1) |  |
| Malignancy^2^ |  |  |  |  |  |
| Yes | 13 | 820.5 | 15.8 (9.2-27.3) | 3.22 (1.79-5.76) | 1.87 (1.02-3.40) |
| No | 86 | 17456.7 | 4.9 (4.0-6.1) | Ref (1) |  |

Abbreviations: aMRR, adjusted mortality rate ratio; ART, antiretroviral therapy; IDU, injection drug use; MR, mortality rate; MRR, mortality rate ratio; MSM, men who have sex with men.

^1^ The aMRR was adjusted for age, transmission mode, CD4 cell count recovery, diabetes mellitus, chronic lung disease, chronic liver disease, malignancy, and calendar time and AIDS-defining event.

^2^ Haematological or solid organ malignancy.

**eTable 7. Incidence rate of first cardiovascular (CV) event, including people living with HIV initiating ART in the study period, surviving the first two years and with available CD4 cell count both at ART initiation and 2 years after.**

|  | CV Events  (n) | Person-years (PY) | IR per 1000 PY (95% CI) | IRR (95% CI) | aIRR (95%CI)^1^ |  |
| --- | --- | --- | --- | --- | --- | --- |
| Total | 103 | 15743.3 | 6.5 (5.4-7.9) |  |  |  |
| Gender |  |  |  |  |  |  |
| Female | 14 | 2440.2 | 5.7 (3.4-9.7) | Ref (1) |  |  |
| Male | 89 | 13303.1 | 6.7 (5.4-8.2) | 1.17 (0.66-2.05) |  |  |
| Age (time-updated) (years) |  |  |  |  |  |  |
| <40 | 12 | 6305.5 | 1.9 (1.1-3.4) | Ref (1) | Ref (1) |  |
| 40-49 | 28 | 5628.1 | 5.0 (3.4-7.2) | 2.61 (1.33-5.14) | 2.30 (1.16-4.55) |  |
| 50-59 | 43 | 2879.0 | 14.9 (11.1-20.1) | 7.85 (4.14-14.88) | 6.11 (3.16-11.82) |  |
| ≥60 | 20 | 930.8 | 21.5 (13.9-33.3) | 11.29 (5.52-23.10) | 7.09 (3.25-15.44) |  |
| Country of birth |  |  |  |  |  |  |
| Spain | 74 | 8314.3 | 8.9 (7.1-11.2) | Ref (1) |  |  |
| Europe | 10 | 1109.0 | 9.0 (4.9-16.8) | 1.01 (0.52-1.96) |  |  |
| Africa | 6 | 688.5 | 8.7 (3.9-19.4) | 0.98 (0.43-2.25) |  |  |
| America | 6 | 3236.4 | 1.9 (0.8-4.1) | 0.21 (0.09-0.48) |  |  |
| Asia | 1 | 150.1 | 6.7 (0.9-47.3) | 0.75 (0.10-5.38) |  |  |
| Transmission mode |  |  |  |  |  |  |
| MSM | 40 | 9430.2 | 4.2 (3.1-5.8) | Ref (1) | Ref (1) |  |
| Heterosexual men | 28 | 2284.8 | 12.3 (8.5-17.7) | 2.89 (1.78-4.68) | 1.94 (1.17-3.24) |  |
| Women | 9 | 2018.8 | 4.5 (2.3-8.6) | 1.05 (0.51-2.17) | 0.85 (0.41-1.77) |  |
| IDU | 16 | 1145.8 | 14.0 (8.6-22.8) | 3.29 (1.84-5.88) | 1.60 (0.82-3.15) |  |
| Unknown/other | 10 | 863.7 | 11.6 (6.2-21.5) | 2.73 (1.37-5.46) | 1.76 (0.85-3.62) |  |
| CD4 cell count at ART initiation |  |  |  |  |  |  |
| Late presenters (CD4 ≤350 cells/µL) | 67 | 9251.5 | 7.2 (5.7-9.2) | 1.31 (0.87-1.96) |  |  |
| Non-late presenters, (CD4 >350 cells/µL) | 36 | 6491.8 | 5.5 (4.0-7.7) | Ref (1) |  |  |
| CD4 cell count 2 years after ART initiation |  |  |  |  |  |  |
| Late presenters, CD4 <200 cells/µL | 8 | 703.4 | 11.4 (5.7-22.7) | 2.24 (1.03-4.91) | 1.04 (0.45-2.38) |  |
| Late presenters, CD4 200-500 cells/µL | 35 | 4452.1 | 7.9 (5.6-10.9) | 1.55 (0.95-2.54) | 0.84 (0.49-1.43) |  |
| Late presenters, CD4 >500 cells/µL | 24 | 4096.0 | 5.9 (3.9-8.7) | 1.16 (0.67-1.98) | 0.92 (0.53-1.59) |  |
| Non-late presenters, CD4 ≤500 cells/µL | 7 | 772.2 | 9.1 (4.3-19.0) | 1.79 (0.78-4.08) | 1.44 (0.62-3.35) |  |
| Non-late presenters, CD4 >500 cells/µL | 29 | 5719.6 | 5.1 (3.5-7.3) | Ref (1) | Ref (1) |  |
| HIV viral load >200 c/ml 2 years after ART initiation |  |  |  |  |  |  |
| Yes | 9 | 1024.3 | 8.8 (4.6-16.9) | 1.37 (0.69-2.71) |  |  |
| No | 91 | 14155.8 | 6.4 (5.2-7.9) | Ref (1) |  |  |
| Calendar time (time-updated) |  |  |  |  |  |  |
| 2005-2009 | 4 | 418.0 | 9.6 (3.6-25.5) | 1.46 (0.54-4.01) | 1.79 (0.64-4.95) |  |
| 2010-2014 | 25 | 3994.0 | 6.3 (4.2-9.3) | 0.96 (0.61-1.51) | 1.09 (0.68-1.73) |  |
| 2015-2021 | 74 | 11331.4 | 6.5 (5.2-8.2) | Ref (1) | Ref (1) |  |
| AIDS defining event in the first 2 years after ART initiation |  |  |  |  |  |  |
| Yes | 20 | 1486.0 | 13.5 (8.7-20.9) | 2.31 (1.42-3.77) |  |  |
| No | 83 | 14257.4 | 5.8 (4.7-7.2) | Ref (1) |  |  |
| Educational level, n (%) |  |  |  |  |  |  |
| None or primary education only | 35 | 3528.7 | 9.9 (7.1-13.8) | Ref (1) |  |  |
| Secondary education | 21 | 4515.8 | 4.7 (3.0-7.1) | 0.47 (0.27-0.81) |  |  |
| University | 12 | 3445.5 | 3.5 (2.0-6.1) | 0.35 (0.18-0.68) |  |  |
| Comorbidities 2 years after ART initiation |  |  |  |  |  |  |
| Diabetes |  |  |  |  |  |  |
| Yes | 8 | 204.1 | 39.2 (19.6-78.4) | 6.41 (3.12-13.2) | 2.74 (1.19-6.30) |  |
| No | 95 | 15539.2 | 6.1 (5.0-7.5) | Ref (1) | Ref (1) |  |
| Arterial hypertension |  |  |  |  |  |  |
| Yes | 13 | 1069.1 | 12.2 (7.0-20.8) | 1.98 (1.11-3.55) | 1.01 (0.52-1.99) |  |
| No | 90 | 14674.3 | 6.1 (5.0-7.5) | Ref (1) | Ref (1) |  |
| Dyslipidaemia |  |  |  |  |  | |
| Yes | 14 | 823.4 | 17.0 (10.1-28.7) | 2.85 (1.62-5.01) | 1.44 (0.77-2.67) | |
| No | 89 | 14920.0 | 6.0 (4.8-7.3) | Ref (1) | Ref (1) | |
| Chronic kidney disease |  |  |  |  |  |  |
| Yes | 3 | 84.9 | 35.3 (11.4-109.6) | 5.53 (1.76-17.45) | 2.14 (0.64-7.12) |  |
| No | 100 | 1558.5 | 6.4 (5.2-7.8) | Ref (1) | Ref (1) |  |
| Chronic lung disease |  |  |  |  |  |  |
| Yes | 4 | 196.5 | 20.4 (7.6-54.2) | 3.20 (1.18-8.69) | 1.01 (0.36-2.88) |  |
| No | 99 | 15546.9 | 6.4 (5.2-7.8) | Ref (1) | Ref (1) |  |
| Chronic liver disease |  |  |  |  |  |  |
| Yes | 21 | 1160.2 | 18.1 (11.8-27.8) | 3.22 (1.99-5.20) | 2.14 (1.25-3.68) |  |
| No | 82 | 14583.2 | 5.6 (4.5-7.0) | Ref (1) | Ref (1) |  |
| Malignancy^2^ |  |  |  |  |  |  |
| Yes | 15 | 700.1 | 21.4 (12.9-35.5) | 3.66 (2.12-6.33) | 2.26 (1.29-3.98) |  |
| No | 88 | 15043.2 | 5.8 (4.7-7.2) | Ref (1) | Ref (1) |  |
| Depression |  |  |  |  |  |  |
| Yes | 16 | 1409.7 | 11.3 (7.0-18.5) | 1.87 (1.10-3.19) | 1.30 (0.75-2.24) |  |
| No | 87 | 14333.6 | 6.1 (4.9-7.5) | Ref (1) |  |  |

Abbreviations: aIRR, adjusted incidence rate ratio; ART, antiretroviral therapy; CVD, cardiovascular disease (includes ischemic heart disease, congestive heart failure, cerebrovascular disease, peripheral vascular disease); IDU, injection drug use; IR, incidence rate; IRR, incidence rate ratio; MSM, men who have sex with men.

^1^ The aIRR was adjusted for age, transmission mode, CD4 cell count recovery, diabetes mellitus, arterial hypertension, dyslipidaemia, chronic kidney disease, chronic lung disease, chronic liver disease, malignancy, depression, and calendar time, and AIDS-defining event.

^2^ Haematological or solid organ malignancy.

**eFigure 2: Kaplan-Meier curve. Survival following a first CV event in PLWH, in late presenters versus non-late presenters (including all patients initiating ART without prior cardiovascular event (CVE)).**

**eTable 8. All-cause mortality from first cardiovascular (CV) event in people living with HIV, including all patients initiating ART without prior CV events, regardless of survival the first two years.**

|  | Deaths after a CVE | MR per 1000 PY (95%CI) | MRR (95%CI) | aMRR^1^ (95%CI) |
| --- | --- | --- | --- | --- |
| Total | 17 | 38.1 (23.7-61.3) |  |  |
| Gender |  |  |  |  |
| Male | 13 | 33.9 (19.7-58.3) | Ref (1) |  |
| Female | 4 | 64.3 (24.1-171.3) | 1.90 (0.62-5.83) |  |
| Age at baseline |  |  | 1.08 (1.03-1.13) | 1.07 (1.02-1.12) |
| Route of HIV transmission,  n (%) |  |  |  |  |
| MSM | 2 | 11.9 (3.0-47.4) | Ref (1) | Ref (1) |
| Heterosexual men | 7 | 59.6 (28.4-125.0) | 5.03 (1.04-24.20) | 3.06 (0.57-16.45) |
| Women | 4 | 143.2 (53.8-381.6) | 12.08 (2.21-65.96) | 8.08 (1.34-48.94) |
| IDU | 2 | 24.8 (6.2-99.2) | 2.09 (0.29-14.86) | 2.72 (0.35-20.89) |
| Unknown | 2 | 38.8 (9.7-155.3) | 3.28 (0.46-23.26) | 2.56 (0.34-19.14) |
| Cardiovascular disease |  |  |  |  |
| Heart failure | 5 | 41.9 (17.4-100.7) | 3.00 (0.58-15.47) |  |
| Ischemic heart disease | 2 | 14.0 (3.5-55.8) | Ref (1) |  |
| Cerebrovascular disease | 8 | 64.7 (32.4-129.4) | 4.63 (0.98-21.83) |  |
| Peripheral vascular disease | 2 | 33.3 (8.3-133.1) | 2.38 (0.34-16.92) |  |
| Calendar time |  |  |  |  |
| 2005-2019 | 3 | 68.8 (25.8-183.3) | 2.05 (0.67-6.30) |  |
| 2015-2021 | 13 | 33.5 (19.5-57.7) | Ref (1) |  |
| AIDS at ART initiation |  |  |  |  |
| Yes | 7 | 98.6 (47.0-206.9) | 3.70 (1.41-9.72) |  |
| No | 10 | 26.7 (14.3-49.5) | Ref (1) |  |
| CD4 cell count at ART initiation |  |  |  |  |
| Late presenters, CD4 ≤350 cells/ µL | 15 | 46.1 (27.8-76.5) | 2.79 (0.64-12.21) | 1.21 (0.25-5.97) |
| Non-late presenters, CD4 >350 cells/ µL | 2 | 16.5 (4.1-66.1) | Ref (1) | Ref (1) |
| CD4 cell count 2 years after ART initiation |  |  |  |  |
| Late presenters, CD4 <200 cells/µL | 2 | 48.8 (12.2-195.1) | 2.45 (0.34-17.36) |  |
| Late presenters, CD4 200-500 cells/µL | 9 | 48.6 (25.3-93.5) | 2.44 (0.53-11.28) |  |
| Late presenters, CD4 >500 cells/µL | 4 | 40.4 (15.2-107.6) | 2.02 (0.37-11.05) |  |
| Non-late presenters, CD4 ≤500 cells/µL | 0 | - |  |  |
| Non-late presenters, CD4 <500 cells/µL | 2 | 19.9 (5.0-79.8) | Ref (1) |  |
| Comorbidity at ART initiation |  |  |  |  |
| Diabetes mellitus |  |  |  |  |
| Yes | 2 | 59.4 (21.9-60.3) | 1.63 (0.37-7.15) |  |
| No | 15 | 36.4 (21.9-60.3) | Ref (1) |  |
| Arterial hypertension |  |  |  |  |
| Yes | 1 | 18.0 (2.5-128.0) | 0.44 (0.06-3.32) |  |
| No | 16 | 40.9 (25.1-66.8) | Ref (1) |  |
| Dyslipidaemia |  |  |  |  |
| Yes | 1 | 18.5 (2.6-131.2) | 0.45 (0.06-3.41) |  |
| No | 16 | 40.8 (25.0-66.6) | Ref (1) |  |
| Chronic kidney disease |  |  |  |  |
| Yes | 0 | - | - |  |
| No | 17 | - | - |  |
| Chronic lung disease |  |  |  |  |
| Yes | 2 |  | 4.07 (0.93-17.81) | 4.33 (0.96-19.53) |
| No | 15 | 34.7 (20.9-57.6) | Ref (1) | Ref (1) |
| Chronic liver disease |  |  |  |  |
| Yes | 3 | 35.7 (11.5-110.6) | 0.92 (0.26-3.21) |  |
| No | 14 | 38.7 (22.9-65.3) | Ref (1) |  |
| Malignancy^2^ |  |  |  |  |
| Yes | 2 | 28.3 (7.1-113.1) | 0.71 (0.16-3.09) |  |
| No | 15 | 40.0 (24.1-66.3) | Ref (1) |  |
| Depression |  |  |  |  |
| Yes | 2 | 33.6 (8.4-134.5) | 0.87 (0.20-3.79) |  |
| No | 15 | 38.8 (23.4-64.3) | Ref (1) |  |

Abbreviations: aMRR, adjusted mortality rate ratio; ART, antiretroviral therapy; CVE, cardiovascular event; IDU, injection drug use; MR, mortality rate; MRR, mortality rate ratio; MSM, men who have sex with men.

^1^ The aMRR was adjusted for age, transmission mode, CD4 cell count at ART initiation, and chronic lung disease.

^2^ Haematological or solid organ malignancy.

**eFigure 3. Kaplan-Meier curve of cumulative survival following a first cardiovascular (CV) event. A: Survival from the first CV event in people living with HIV (PLWH) surviving the first two years after ART initiation. B: Survival from the first CV event in PLWH surviving the first two years after ART initiation, in late presenters versus non-late presenters.**

**eTable 9. Modified Charlson comorbidity score used in the study.**

| Comorbid conditions | Score |
| --- | --- |
| Myocardial infarction* | 1 |
| Congestive heart failure* | 1 |
| Peripheral vascular disease* | 1 |
| Cerebrovascular disease* | 1 |
| Dementia | 1 |
| Chronic pulmonary disease | 1 |
| Diabetes | 1 |
| Liver disease | 1 |
| Renal disease | 2 |
| Solid organ malignancy | 2 |
| Haematological malignancy | 2 |

*PWH with prior CV events (ischemic heart disease, congestive heart failure, cerebrovascular disease, or peripheral vascular disease) were excluded from the analysis.
